# Early detection of health problems in potentially frail community-dwelling older people by general practices - project [G]OLD: design of a longitudinal, quasi-experimental study

**DOI:** 10.1186/1471-2318-13-7

**Published:** 2013-01-18

**Authors:** Mandy MN Stijnen, Inge GP Duimel-Peeters, Maria WJ Jansen, Hubertus JM Vrijhoef

**Affiliations:** 1 Department of General Practice, School for Public Health and Primary Care (CAPHRI), Faculty of Health, Medicine and Life Sciences, Maastricht University Medical Centre, P.O. Box 616, 6200 MD, Maastricht, The Netherlands; 2 Department of Patient & Care, Maastricht University Medical Centre, P.O. Box 5800, 6202 MD, Maastricht, The Netherlands; 3 Public Health Service South-Limburg, School for Public Health and Primary Care (CAPHRI), P.O. Box 2022, 6160 HA, Geleen, The Netherlands; 4 Tilburg University, Scientific Centre for Care and Welfare (TRANZO), Tilburg, The Netherlands; 5 National University of Singapore, Saw Swee Hock School for Public Health, Singapore, Singapore

**Keywords:** Frailty, Older people, Comprehensive geriatric assessment, Home visit, General practice, Quasi-experimental design

## Abstract

**Background:**

Due to the ageing of the population, the number of frail older people who suffer from multiple, complex health complaints increases and this ultimately threatens their ability to function independently. Many interventions for frail older people attempt to prevent or delay functional decline, but they show contradicting results. Recent studies emphasise the importance of embedding these interventions into existing primary care systems and tailoring care to older people’s needs and wishes. This article presents the design of an evaluation study, aiming to investigate the effects and feasibility of the early detection of health problems among community-dwelling older people and their subsequent referral to appropriate care and/or well-being facilities by general practices.

**Methods/Design:**

A longitudinal, quasi-experimental study is designed comparing 13 intervention practices with 11 control practices. General practices select eligible community-dwelling older people (≥ 75 years). Practice nurses from intervention practices (1) visit older people at home for a comprehensive assessment of their health and well-being; (2) discuss results with the GP; (3) formulate – if required – a care and treatment plan together with the patient; (4) refer patient to care and/or well-being facilities; and (5) monitor and coordinate care and follow-up. Control practices provide usual care and match the intervention practices on the presence of different primary care professionals within the practice. Primary outcome measures are health-related quality of life and disability. Additionally, attitude towards ageing, care satisfaction, health care utilisation, nursing home admission and mortality are measured. Some outcomes are assessed by means of a postal questionnaire (at baseline and after 6, 12, and 18 months follow-up), others through continuous registration over the 18-month period. A profound process evaluation will provide insight into barriers and facilitators for implementing the intervention protocol within general practices from both the patient and caregiver perspective.

**Discussion:**

The proposed approach requires redesigning care delivery within general practices for accomplishing appropriate care for older people. A quasi-experimental design is chosen to closely resemble a real-life situation, which is desirable for future implementation after this innovation proves to be successful. Results of the effect and process evaluation will become available in 2013.

**Trial registration:**

The Netherlands National Trial Register NTR2737

## Background

Ageing of the population poses challenges to health care systems as the number of frail older people who suffer from complex and/or multiple (chronic) health complaints increases [1,2]. A failure to detect health complaints among older people in time may cause unnecessary neediness and may threaten their ability to function independently.

Strategies comprising early identification of older people at risk of poor health and early intervention should prevent or postpone the onset of functional decline and maintain independent living [3]. In the last decades, there has been an increased focus worldwide on the development of preventive home visitation programmes to support older people to grow old at home and to prevent or delay institutionalisation.

There is still an ongoing debate whether these preventive home visits should be part of regular care for older people. Numerous systematic reviews have been published [4-11], attempting to determine the effectiveness of preventive home visits, but the results remain inconclusive. Discrepancy in the results is caused, among others, by differences in the selection of the target population, intensity and duration of the intervention (i.e., number of follow-up visits), or domains included in the multidimensional assessment of older people’s health status [12]. Thus, the question remains which components of preventive home visits are effective and for which population they are beneficial [13]. Most studies to date employ a randomised design for establishing the success of preventive home visits, thereby hindering close resemblance to a real-life situation and restricting the external validity of findings.

Recent publications stress the importance of integrating preventive interventions for older people into existing care systems [10,14,15]. For example, Van Hout and colleagues [14] attribute the absence of a preventive effect of home visits to the fact that they were not integrated within primary care practices. In our current approach, instead of solely integrating, we aim to redesign care delivery within primary care practices by applying components of the Chronic Care Model (CCM). This comprehensive framework has proven to lead to improved patient care and better health outcomes when changing routine delivery of care through improvements in six interrelated components (further details are provided in the Methods section) [16,17]. In addition, elements of the Guided Care model are incorporated in our approach [18]. Guided Care used the CCM to identify successful innovations in chronic care that can be applied in primary care to achieve optimal outcomes in people with chronic diseases and complex care needs.

General practices seem to be the ideal setting for realising preventive care facilities for older people, because of their geographical proximity to older people, knowledge of the patient’s medical history, relationship of trust between doctor and patient, and access to a range of multidisciplinary health care and well-being facilities in the person’s neighbourhood. However, general practitioners (GPs) often do not have a complete overview of the health status and functioning of older people [19,20]. A Dutch study among randomly selected older patients revealed that 34% of recorded health problems during a home visit were unknown to GPs (mostly psychosocial or physical complaints, such as depression and urine incontinence) [19]. Similarly, Alessi and co-workers [21] reported that three-quarter of the visited older people had at least one major health problem identified that was previously unknown. This suggests that a comprehensive geriatric assessment in the home setting yields important information about previously undetected health problems and this might be particularly beneficial for the *apparently* healthy older people.

It is equally important that older people themselves are aware of their own (unmet) health needs, as this appears to be supportive for maintaining independent living [22]. It seems that older people tend to discard certain health problems or complaints as inevitable aspects of ageing, such as in the case of urinary incontinence [23], they forget about the occurrence of certain events, such as in reporting falling incidents [24], or they may fail to recognise the significance of symptoms or complaints [25]. A multidimensional assessment may create awareness of these (unmet) needs or problems. After health problems and complaints are identified, care facilities should be tailored to older patient’s needs and preferences [26-28] and active involvement of older people in decision-making concerning their need for care services is encouraged [29].

In conclusion, we hypothesise that a multidimensional comprehensive geriatric assessment of the health and well-being of community-dwelling older people by general practices and subsequent individualised care and follow-up (if required) will lead to improved health-related quality of life and reduced disability compared to usual care (i.e., reactive care to older people who present themselves with health problems or complaints). Furthermore, we hypothesise that this approach will be feasible from both the patient and caregiver perspective. In the current paper, the design of an evaluation study is presented aiming to investigate the effects of identifying health problems and complaints among potentially frail community-dwelling older people at an early stage and, if necessary, their follow-up within care and/or well-being facilities. Parallel to the effect study, a process evaluation will provide insight into the barriers and facilitators for implementing the proposed approach within general practices.

## Methods/Design

### Study design and setting

**Figure 1:**
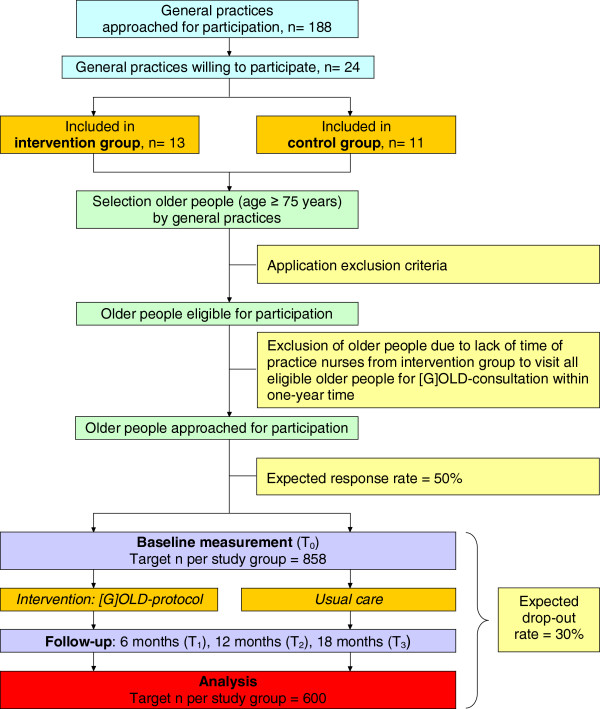
Flow chart study design and measurements.

**Figure 2:**
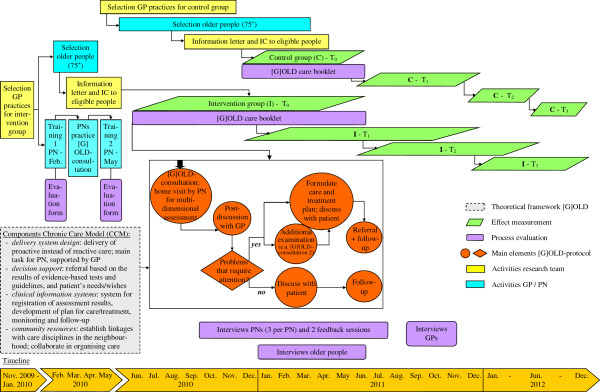
**Schematic overview study protocol. **Note: PN = practice nurse; GP = general practitioner; IC = informed consent.

The longitudinal, quasi-experimental study is performed in three regions in the south of the Netherlands: Maastricht-Heuvelland (8.5% ≥ 75 years), Parkstad (8.7% ≥ 75 years), and Midden-Limburg (7.5% ≥ 75 years). They are particularly interesting because the ageing of the population is more pronounced here (nationwide 7.0% ≥ 75 years). General practices in these regions were invited to participate in the evaluation study. Participating general practices randomly selected community-dwelling people aged 75 years and older from the GP Information System. Older people within intervention practices are visited at home by the practice nurse for a multidimensional assessment followed by individualised care, the so-called [G]OLD-protocol: ‘Getting OLD the healthy way’. Older people from control practices receive usual care (i.e., reactive care instead of proactive care). Effects on outcome measures are assessed at baseline (T_0_) and after 6-months (T_1_), 12-months (T_2_), and 18-months (T_3_) follow-up. Parallel to the effect study, a process evaluation is performed. Figure 1 presents a flow chart of the study design and measurements. A more complete overview of the study protocol, including a time schedule, is provided in Figure 2.

The Medical Ethical Committee (MEC) of the Maastricht University Medical Centre (MUMC+) judged this evaluation study as not needing formal ethical approval. Nevertheless, the MEC granted their approval for our study protocol and informed consent documents.

### Selection of general practices

General practices (n = 21) who visited older people at home as part of our pilot study [30] were excluded from participation to prevent contamination of prior experience. We approached 188 general practices in the three regions for participation in this study. Practices in Midden-Limburg were only invited to participate in the control group, since insufficient general practices from the other two regions were willing to participate in the control group. GPs who indicated to be actively engaged in or are planning to start with the identification and follow-up of frail older people in a systematic way were ineligible to participate (n = 12 practices). The availability of a practice nurse who has time for care for older people is a prerequisite for intervention practices. Practice nurses work in general practices, and provide screening, treatment, care and education mainly to patients with chronic diseases and older people.

Reasons of general practices for non-participation were: no time (e.g., due to other priorities, staff changes or participation in other research projects) (35.0%), no interest to participate in the present study (31.7%), interested in [G]OLD-intervention but not in research (19.5%), or no reason was mentioned (13.8%).

Fourteen general practices agreed to participate as intervention practice and 13 general practices consented to participate in the control group. Control practices were matched to intervention practices based on the presence of primary care professionals within general practices to ensure comparability at baseline. We assume that close proximity of various primary care disciplines facilitates collaboration in organising and/or delivering appropriate care to older people [31]. After the recruitment phase, one intervention practice and two control practices dropped out due to a lack of time to select older people eligible for participation. As a result, 24 general practices were included in this study.

### Target population

The target population are the apparently healthy, community-dwelling older people aged 75 years and older. Although the age criterion causes much controversy, especially from the age of 75 years on the prevalence of frailty increases markedly [32]. This enables us to find sufficient eligible older people for participation. Furthermore, some authors suggest that preventive home visits are most beneficial for people aged 75 years and older [33,34]. We excluded people who are not living independently, those on a waiting list for admission to a nursing home or home for older people, those under close medical supervision (chemotherapy, chronic haemodialysis or other therapies posing a high burden on the person), and the terminally ill. Practice nurses’ available working hours for care for older people determined the maximum number of older people that each of them was able to visit within one year's time. This, together with the size of the patient population aged 75 years and older, determined the number of older people per intervention practice approached for participation. In control practices, all eligible older people aged 75 years and over were approached.

We invited older people for participation by means of an information letter and consent form. We performed telephonic reminders in the intervention group and postal reminders after four weeks in the control group to those who did not respond to the first mailing.

### Procedure

Although the independent effects of components of preventive home visitation programmes are difficult to disentangle, previous research has suggested elements that at least should be included, such as a comprehensive geriatric assessment, a concrete care plan and multiple follow-up contacts [7,8,29]. We redesigned care delivery for older people by general practices by focussing on several evidence-based elements of the Chronic Care Model (CCM) and the Guided Care model (for details, see Figure 2). Applying both models has led to the development of the [G]OLD-protocol, which is explained in more detail below. Our pilot study provided preliminary evidence of the feasibility of the [G]OLD-protocol for general practices [30].

#### Training

Practice nurses from intervention practices received two days of training before the start of the study to provide them with the necessary knowledge and skills for executing all elements of the [G]OLD-protocol. In this way, we also attempted to equalise the level of knowledge and skills between practice nurses regarding care for older people. Central elements of the training included acquiring communication skills, gaining knowledge about frequently occurring health problems among older people, gaining knowledge about health services for older people, and learning how to assess older people’s physical, psychological, mental and social functioning by means of a multidimensional instrument. In between the two training sessions, each practice nurse performed exactly five home visits among randomly chosen older people (≥ 75 years) during a try-out phase. During the intervention period, sessions were organised for asking questions and exchanging experiences, and practice nurses received additional support by a coach specialised in geriatric care.

#### Home visit - comprehensive geriatric assessment

The practice nurse invites older people for a home visit successively within a one-year time period. Before the visit, the practice nurse makes a print out of the person’s medication list and medical history for relevant details or major events to be aware of.

During the visit, the practice nurse uses the [G]OLD-instrument: a structured, comprehensive geriatric assessment to assess the person’s physical, psychological, mental and social functioning, as well as lifestyle and medication use (see Table 1). This instrument is specifically developed for and tested among the apparently healthy community-dwelling older people aged 75 years and older in a pilot study [30]. Suggestions made during the pilot phase helped to improve the [G]OLD-instrument for application in the current longitudinal, quasi-experimental study. In general, the instrument assists the practice nurse in uncovering (early signs of) potential health problems or needs that may prevent older people, now or in the near future, from maintaining independent living. Although the instrument follows a structured format, it can be applied in a flexible way. For each test included, evidence-based cut-off points and guidelines are presented to assist in deciding about the presence or absence of health problems or needs.

**Table 1:** **Topics included in the **[**G**]**OLD comprehensive geriatric assessment instrument**

	**Basic assessment –part one**	**Additional assessment** – **part two**
Physical functioning and lifestyle	Disability in ADL and IADL; need for assistance in ADL and/or IADL; incontinence; mobility; falls; vision and hearing problems; BMI (height and weight); malnutrition; blood pressure; physical activity; smoking; alcohol use	N/A
Psychological functioning	Cognition; anxiety; depression; personality disorders	Cognition; depression; personality disorders
Social functioning	Receiving and providing informal care; loneliness; social participation	N/A
Additional	General perception of health and quality of life; medication use; financial situation; health care utilisation; observation of living environment; physical, psychological and behavioural signals	N/A

Note: N/A means not applicable.

Crucial during the visit is establishing a relationship of trust, listening to the needs and wishes of the older person, and allowing the person time to talk [29]. If necessary, the practice nurse may also provide information or advice. Sometimes it is necessary to perform an additional examination to obtain a more accurate estimation of the presence of problems. Therefore, more elaborate tests on the themes cognition, depression and personality disorders are incorporated in the [G]OLD-instrument part 2 which can be conducted during the first visit or during a second visit, depending on the older person’s preference.

After the home visit, the practice nurse registers the results of the [G]OLD-instrument in the electronic patient file.

#### Post-discussion GP and formulating care and treatment plan

The practice nurse discusses the results of the home visit with the GP. The results of the [G]OLD-instrument, as well as the patient’s needs and wishes, determine whether follow-up actions regarding certain problems are needed. These actions may consist of additional diagnosis, preventive care or advise, treatment in primary health care or referral to other care and/or well-being facilities as much as possible in the older person’s neighbourhood. The practice nurse formulates a provisional individualised care and treatment plan. This plan is discussed with the patient, whose input and wishes lead to a final care and treatment plan, which is registered in the electronic patient file.

#### Referral and follow-up

The practice nurse arranges and coordinates care for the older person as formulated in the final care and treatment plan and monitors the follow-up. The need for and frequency of follow-up contacts strongly depends on the type of problems or complaints that deserve attention according to the care and treatment plan. Hence, no fixed number of contacts per older person is determined on forehand. The practice nurse indicates in the care and treatment plan at what date a specific problem or complaint will be re-evaluated. Then, at each follow-up contact, the need for additional follow-up contacts is determined and, if necessary, the care and treatment plan is adjusted. Notably, these follow-up contacts may also take place with other care providers to whom older people are being referred.

If follow-up actions are not required or they are not desirable from the patient’s point of view, the practice nurse discusses with both the GP and the older person how they will proceed from that moment on. It is important that general practices should prevent to lose sight of their older patients after this initial assessment. The home visit is not meaningless when no specific problems are identified, as it helps to gain knowledge about the patient in case future health problems occur (e.g., falling incidents). Furthermore, the bond created with the practice nurse increases the likelihood that older people will approach their general practice in case of any future problems or complaints. Older people who do not receive follow-up contacts will remain part of the study population to ensure comparability with the control group and they will be analysed as a sub-group.

### Measures and data collection

The primary outcome measures in this study are health-related quality of life measured by the RAND-36 [35,36] and disability in activities of daily living (ADL, including mobility) and instrumental activities of daily living (IADL), assessed using the Groningen Activity Restriction Scale (GARS) [37]. Both instruments appear to be valid, reliable and suitable for self-completion in older people [38,39]. These outcomes, together with the secondary outcome attitude towards ageing (subscale attitude toward ageing from the PGC Morale Scale) [40] are included in a questionnaire send to older people by postal mail at baseline, 6-months, 12-months and 18-months follow-up. The baseline questionnaire also gathers data about socio-demographic variables (i.e., age, gender, ethnicity, educational level, marital status, household composition) to provide insight into characteristics of the target population. Assistance is provided to older people who are unable to self-complete the questionnaires or those with many missing items (mostly people with poor physical or mental health).

Additional secondary outcomes are admission to a nursing home or home for older people, health care utilisation, and mortality. General practices register these outcomes continuously during the study period in the GP Information System and data are extracted for each patient after 18-months follow-up. Furthermore, health care utilisation is also recorded in the [G]OLD care booklet. Older people receive this booklet at baseline and are requested to take it with them to each contact with professional health care providers for 18 months. In this booklet, patients and/or care providers indicate the reason for the contact, type of health problems or complaints, and follow-up activities. Table 2 presents all outcome measures, their operationalisation and timing of data collection.

**Table 2:** **Measures**, **operationalisation and timing of data collection**

**Measures**	**Operationalisation**	**No**. **of items**	Range score^*^	**Timing data collection**^†^
*Primary outcomes*				
Health-related quality of life	RAND-36 [35,36]	36	N/A	T_0_, T_1_, T_2_, T_3_
Disability	GARS [37]	18	18–72	T_0_, T_1_, T_2_, T_3_
	IADL	11	11-44	
	ADL	7	7–28	
*Secondary outcomes*				
Attitude towards ageing	Subscale attitude toward own ageing - PGC Morale Scale [40]	5	0–5	T_0_, T_1_, T_2_, T_3_
Health care utilisation	Number of contacts with different health care providers (i.e., GP consultations, hospital admission)	3	N/A	T_0_
		N/A	N/A	CR_GP and CR_E
Admission to nursing home or home for older people	Number of admissions and time to admission from T_0_ to T_3_	N/A	N/A	CR_GP
Mortality	Number of deaths from T_0_ to T_3_	N/A	N/A	CR_GP

* Underlined scores indicate the most favourable scores. N/A means not applicable.

† T_0_ = postal questionnaire at the start of the study; T1 = postal questionnaire at 6-months follow-up; T2 = postal questionnaire at 12-months follow-up; T3 = postal questionnaire at 18-months follow-up; CR_GP = continuous registration during study in GP’s Information System; CR_E = continuous registration during study by older people in [G]OLD care booklet.

### Process evaluation

A thorough process evaluation is conducted aiming to investigate to what extent the different components of the [G]OLD-protocol are implemented within general practices as planned (e.g., barriers and facilitators for implementation) and the feasibility of the protocol for both patients and caregivers. Ultimately, the results may provide information for further implementation of [G]OLD within general practices. Qualitative and quantitative process data are collected with either formative or summative purposes among GPs, practice nurses and older people according to the comprehensive and systematic approach proposed by Saunders and colleagues [41] (for details, see Table 3).

**Table 3:** Data collection as part of the process evaluation

**Components**	**Operationalisation**	**Data collection Tools/Procedures**
*Fidelity* (*quality*)	Extent to which the [G]OLD-protocol was implemented as planned	Evaluation form training PNs
		Individual interviews PNs, GPs and older people
		Feedback sessions PNs
		[G]OLD-instrument and care and treatment plan
*Dose delivered* (*completeness*)	Extent to which all aspects of [G]OLD-protocol are delivered to general practices and older people	Evaluation form training PNs
		Individual interviews PNs, GPs and older people
		Feedback sessions PNs
		[G]OLD-instrument and care and treatment plan
*Dose received* (*exposure*)	Extent to which PNs, GPs and older people actively engage in, interact with and are receptive to aspects of [G]OLD-protocol	Individual interviews PNs, GPs and older people
		Feedback sessions PNs
		[G]OLD care booklet for older people
*Dose received* (*satisfaction*)	Overall opinion of PNs, GPs, and older people about [G]OLD	Evaluation form training PNs
		Individual interviews
		Feedback sessions PNs
		[G]OLD care booklet for older people
*Reach* (*participation rate*)	Proportion of intended target population that participates in and completes the intervention:	Continuous registration by general practices and researchers
	(1) registration number and reasons for non-response and drop-out; (2) opinion PNs and GPs about reach	Individual interviews with PNs and GPs
*Context*	Environmental barriers and facilitators that influence implementation [G]OLD, continued involvement in [G]OLD, and/or study outcomes	Individual interviews with PNs and GPs
		Notes researchers and project leaders

Note: PN = practice nurse; GP = general practitioner.

The experience of practice nurses with the [G]OLD-protocol was assessed three times during one year (intervention period) by means of individual interviews. Results of these interviews were fed back to all practice nurses together during feedback sessions after six months and after one year (end of the intervention period). Additionally, GPs were individually interviewed at the end of the intervention period to assess their experiences with the implementation of [G]OLD within their general practice. One older person per general practice was selected for in-depth interviews about their experiences and satisfaction with all aspects of the [G]OLD-protocol, approximately one month after the [G]OLD-consultation took place. Furthermore, older people can register their satisfaction with contacts with professional care givers in the [G]OLD-care booklet. Finally, time required for the home visit, results of the tests performed and preliminary advise given to people during the home visit are registered by the practice nurse on the [G]OLD-instrument. Details about referral to care and/or well-being services are written down in the care and treatment plan. Members of the research team checked monthly during the intervention period to what extent the [G]OLD-instrument and the care and treatment plan were completely filled out. Practice nurses registered the patient’s follow-up within the chain of care in the electronic patient file from which relevant data can be extracted after 18-months follow-up.

### Sample size considerations

The sample size calculation is based on the primary outcome measure health-related quality of life (subscale ‘general health perception’) as measured by the RAND-36 [35,36]. We aim to demonstrate a clinically relevant difference between the mean change score of the intervention and control group of 5.0 on the transformed subscale. This implies a standardised effect size of 0.24 (given SD = 21.2). Based on this and applying a significance level (α) of 0.05 and a power of 0.90, the minimally required number of participants is 564 (n = 282 per study group) using an independent samples t-test (two-sided). However, calculations that take into account the interdependency of the measurements within a cluster (i.e., general practice) and correct for the cluster effect result in a required sample size of 1,200 older people (n = 600 per study group).

We expected a response rate of 50% on the information letter and consent form sent to eligible older people for participation and a drop-out rate of 30%. Accounting for drop-out, we planned to enrol 858 older people per study group to have a sufficient number of participants per group (600 older people) at the end of 18 months follow-up (see also Figure 1). Because of the expected response rate of 50%, we planned to approach at least 1,716 community-dwelling older people per study group for participation.

Since the amount of home visits that is performed depends on the PNs available time, we expected a variation in cluster sizes. This is compensated for by sampling 25% more clusters (i.e., general practices) [42].

### Statistical analyses

We compute descriptive statistics to describe the characteristics of the target population and general practices and to investigate comparability of study groups at baseline. Relevant statistical tests (e.g., t-test, chi-square, analysis of variance, regression analysis) will be applied to analyse effects on primary and secondary outcome measures (level of significance is 0.05; two-tailed). Data will be analysed according to the intention-to-treat principle. In all analyses, there will be a correction for possible baseline differences between participants or general practices. In addition, we will perform sub-group analyses to investigate whether certain groups of older people benefit more from the [G]OLD-protocol than other groups. We will use the software package SPSS for Windows, version 17.0, for all statistical analyses.

Data gathered as part of the process evaluation will be analysed using descriptive techniques, such as calculating scores (e.g., number of drop-outs), narrative description of procedures, and identifying themes in the interviews.

## Discussion

In the present paper, the design of a longitudinal, quasi-experimental study is presented to investigate the effects of the early detection of health problems among community-dwelling older people and their subsequent follow-up within the chain of care by general practices. In contrast to existing studies, we purposefully chose for a quasi-experimental design. Although randomised controlled trials are widely accepted as the “gold standard” for evaluating the effectiveness of interventions, they create artificial situations that may hinder the translation of research findings into practice [43-45]. Moreover, the study may suffer from the uncertain commitment of the people delivering the intervention (in this case the general practice’s staff) to the changes to be made. Routinisation of working methods in daily practice must take place to ensure sustainability of the [G]OLD-protocol [46], which is more difficult to realise within a randomised design. Our combination of an effect study and a thorough process evaluation should provide sufficient information with respect to the feasibility and external validity of the [G]OLD-protocol within general practices.

Furthermore, we predominantly used the Chronic Care Model for redesigning primary care practice as applying elements of this framework appears to lead to improved patient care and better health outcomes among patients [17]. We additionally expect that the multidimensional [G]OLD-instrument will be of added value in providing a comprehensive overview of the older person’s health status, compared to intervention programs that only focus on a limited number of tests or questions in only one or two domains.

Challenges faced during the intervention period are managing internal and external factors (e.g., changes in the general practice’s policy or reimbursement of medical expenses by insurance companies) to allow for continued and adequate implementation. Furthermore, considering the current interest of general practices in care for older people, general practices who participate in the control group are closely monitored until the end of the follow-up period to find out if they implement any activities that are similar to practices applying the [G]OLD-protocol. They may undertake initiatives that improve their care for older people and this may distort the intervention effect. Also, several factors may influence the extent to which general practices are successfully redesigned, such as the influence of existing routines and the care providers own clinical opinion (or “gut feelings”) on medical decision-making and referring older patients to adequate care and/or well-being facilities. Although we provided the necessary guidelines and recommendations to facilitate this process, GPs and practice nurses may not have ignored their own medical expertise in deciding about the diagnosis of health problems and/or referral and follow-up. The process evaluation will provide insight in the extent to which general practices redesign their care delivery to older people according to the [G]OLD-protocol. Results of the effect and process evaluation will become available in 2013.

## Abbreviations

ADL: Activities of Daily Living; BMI: Body Mass Index; CCM: Chronic Care Model; GARS: Groningen Activity Restriction Scale; [G]OLD: Getting OLD the healthy way; GP: General Practitioner; IADL: Instrumental Activities of Daily Living; n: Sample size; PGC Morale Scale: Philadelphia Geriatric Center Morale Scale; RAND-36: Research and Development-36; SD: Standard Deviation.

## Competing interests

The authors declare that they have no competing interests.

## Authors’ contributions

ID as main applicant and MJ as project leader were involved in writing the grant proposal for the current study. All authors participated in the design of the study. MS was responsible for the recruitment of general practices and older people in this study. MS drafted the manuscript with input from the other authors. All authors read, commented on and approved the final manuscript.

## Pre-publication history

The pre-publication history for this paper can be accessed here:

http://www.biomedcentral.com/1471-2318/13/7/prepub
